# Association between gastrointestinal symptoms and specialty care utilization among colon cancer survivors: a cohort study

**DOI:** 10.1007/s00384-024-04685-w

**Published:** 2024-08-14

**Authors:** Anya L. Edwards, Karen Trang, Irina V. Tolstykh, Erin L. Van Blarigan, Katherine Van Loon, Angela Laffan, Dalila Stanfield, Paige Steiding, John Neuhaus, Chloe E. Atreya, Sorbarikor Piawah, Alan P. Venook, Madhulika G. Varma

**Affiliations:** 1 Department of Surgery, University of California, San Francisco, San Francisco, CA USA; 2 Department of Epidemiology and Biostatistics, University of California, San Francisco, San Francisco, CA USA; 3 Department of Urology, University of California, San Francisco, San Francisco, CA USA; 4 Division of Hematology/Oncology, Department of Medicine, University of California, San Francisco, San Francisco, CA USA; 5 Helen Diller Family Comprehensive Cancer Center, University of California, San Francisco, San Francisco, CA USA

**Keywords:** Colon cancer survivorship, Healthcare utilization, Quality of life, Gastrointestinal symptoms

## Abstract

**Purpose:**

Persistent gastrointestinal (GI) symptoms are frequently experienced by colon cancer survivors and may help identify patients with higher utilization of healthcare services. To assess the relationship between GI symptoms and specialty care utilization among colon cancer survivors.

**Methods:**

A prospective longitudinal cohort study at an academic medical center of 126 adults surgically treated for stage I–IV colon cancer between February 2017 and June 2022. Participants reported GI symptoms through the EORTC QLQ-C30 and QLQ-CR29 at enrollment and as frequently as every 6 months for 5 years. Main outcome measures were visits, telephone encounters, and secure messages with a medical provider within specialty oncology clinics within 6 months after each survey completion. Generalized linear mixed regression model for repeated measurements with random trajectory for each participant was performed to estimate the associations between symptoms and healthcare use. Models were adjusted for demographics, clinical and surgical factors, and timing in relation to onset of the COVID-19 pandemic.

**Results:**

In the 6 months after each survey time point, patients averaged 1.2 visits, 0.5 telephone encounters, and 3.2 patient-initiated messages. In adjusted models, those with any abdominal pain (RR 1.45; *p* = 0.002), buttock pain (RR 1.30; *p* = 0.050), or increased stool frequency (RR 1.26; *p* = 0.046) had more clinic visits in the following 6 months than those without these symptoms. Including these three symptoms in one model revealed that only abdominal pain was statistically significantly associated with increased clinic visits (RR 1.36; *p* = 0.016). Patients with any blood or mucus in stool (RR 2.46; *p* = 0.009) had significantly more telephone encounters, and those with any abdominal pain (RR 1.65; *p* = 0.002) had significantly more patient-initiated messages than those without these symptoms.

**Conclusions:**

Our findings identify GI symptoms associated with increased use of oncologic specialty care among colon cancer survivors, with abdominal pain as an important predictor of utilization.

**Implications for cancer survivors:**

Early identification and anticipatory management of colon cancer survivors experiencing abdominal pain may decrease healthcare utilization.

**Supplementary Information:**

The online version contains supplementary material available at 10.1007/s00384-024-04685-w.

## Introduction

Approximately 18 million Americans live with a prior cancer diagnosis. This number is expected to increase due to population growth and improved survival from early detection and treatment [1]. Accordingly, healthcare utilization and associated costs are expected to rise, with cost of cancer care nationally projected to exceed $245 billion by 2030 [2]. Given the limited healthcare system capacity and unsustainable cost increases, identifying and addressing drivers of avoidable healthcare utilization are critical. Additionally, compared to patients without a cancer history, cancer survivors have more financial hardship and bankruptcy which is associated with more emergency department (ED) visits, inadequate preventative care, and worse health-related quality of life (QoL) [3–5]. Identifying factors associated with avoidable healthcare utilization among cancer survivors may reveal opportunities to support these patients and minimize personal and system-wide healthcare costs.

Colorectal cancer is one of the most prevalent cancers in the United States, with over 1.4 million people living with a prior diagnosis [1]. National medical and prescription drug expenditures for these patients are second highest of all cancers, exceeded only by breast cancer [3]. Among colorectal cancer survivors, increased healthcare utilization is associated with comorbidities such as cardiovascular disease and diabetes, disease-specific factors such as tumor location and stage, and demographics such socioeconomic status [6–9]. While these reflect general drivers of high healthcare utilization, patient symptoms are more dynamic and may provide a real-time indication of a patient’s likelihood of future healthcare use. Indeed, fatigue, weight concerns, and sore peri-anal or peri-stoma skin have been associated with increased clinic visits [9, 10]. However, studies before the COVID-19 pandemic do not reflect the shift toward telemedicine, which now represents a significant proportion of healthcare delivery [11, 12]. Moreover, prior studies included patients with both colon and rectal cancer, which have different treatment guidelines, surveillance approaches, symptom profiles, and morbidities.

Prior studies have shown that survivors of colorectal cancer often experience significant gastrointestinal (GI) symptoms, with up to half reporting fecal incontinence or bloating and one-third reporting constipation, diarrhea, and abdominal pain [13]. In some sub-populations, such as women, over 80% report experiencing persistent GI symptoms [14]. Furthermore, those that experience GI symptoms, especially of high severity, experienced worse quality of life, body image, and more psychological distress, potentially contributing toward seeking additional medical care [14].

Our study sought to build on prior work by assessing the relationship between self-reported GI symptoms and healthcare utilization in colon cancer survivors [14, 15]. For our analyses, we used self-reported symptom data collected at multiple time points and ascertained use of multiple modalities of care (in-person and virtual clinic visits, telephone encounters, and patient messaging) at repeated time points through linkage to electronic health records. By better understanding who is at-risk for high use of healthcare services, providers and hospital systems can better support these patients.

## Materials and methods

### Patients

This prospective longitudinal cohort study included adults surgically treated for stage I–IV colon cancer who enrolled at variable time points after their surgery in the open Lifestyle and Outcomes after Gastrointestinal Cancer (LOGIC) study between February 2017 and June 2022. Adults receiving care at University of California, San Francisco (UCSF) with a prior gastrointestinal (colon, rectal, or anal) cancer diagnosis who are able to complete surveys in English are eligible to participate in LOGIC. Patients receive an invitation to enroll in LOGIC when referred to the UCSF GI Oncology Survivorship Clinic, and starting in 2020, invitations are also sent via secure messaging in the patient portal or via patient letters [15]. The cohort analyzed in this study included only those with a diagnosis of colon cancer.

Participating patients self-reported demographic (e.g., age, gender, race, and ethnicity), social (e.g., living arrangement), health behaviors (i.e., physical activity, weight, and diet), and QoL data via online questionnaires at enrollment. Subsequently, online QoL surveys were administered every 6 months. Clinical, surgical, and tumor characteristics, as well as annual changes in clinical status (e.g., local recurrence, new metastasis), were obtained by study personnel via electronic medical record (EMR) review.

As of July 2022, 162 patients with a prior colon cancer diagnosis enrolled in LOGIC. Individuals who did not get colon cancer care at UCSF (*n* = 9), those who had surgery for their colon cancer more than 10 years before enrollment (*n* = 11), and those who did not provide any QoL information (*n* = 16) were excluded. After exclusions, 126 patients were eligible for our study, with a maximum follow-up time of 5 years. Demographic, clinical, disease, and treatment characteristics were not statistically significantly different between the 126 patients who met eligibility criteria and the 36 patients who were excluded (data not shown).

UCSF’s institutional review board approved the study. All study participants signed an informed consent statement in accordance with federal and institutional guidelines. We adhered to the recommended STROBE reporting guidelines.

### QoL questionnaires

The 29-item European Organization for Research and Treatment of Cancer (EORTC) Quality of Life Core Questionnaire (QLQ-CR29) is a health-related QoL survey for colorectal cancer patients, designed to complement the 30-item Quality of Life Core Questionnaire (QLQ-C30) for cancer patients [16, 17]. The QLQ-C30 incorporates nine multi-item scales: five functional scales (physical, role, cognitive, emotional, and social), three symptom scales (fatigue, pain, and nausea and vomiting), and a global health and QoL scale. Several single-item symptom measures are also included [17]. The QLQ-CR29 contains four subscales (urinary frequency, blood and mucus in stool, stool frequency, and body image) and 19 single items [16]. Both surveys asked patients to rate extent of symptoms during the past week(s) on a 4-point Likert scale, from 1 = “not at all” to 4 = “very much.”

### Exposures

Primary exposures were GI symptoms from the QLQ-C30 and QLQ-CR29 surveys. The QLQ-C30 includes self-reported constipation and diarrhea. The QLQ-CR29 includes two subscales (blood or mucus in stool, stool frequency) and five individual symptoms (abdominal pain, buttock pain, bloating, flatulence, fecal incontinence, and sore skin around stoma or anal area).

The blood or mucus in stool and stool frequency subscales each include two questions. For the former, patients are asked to indicate whether blood or, separately, mucus was present in stool during the past week. For the latter, patients are asked whether “frequent” bowel movements (or bag changes if stoma bag present) occurred during the day and, separately, at night. For the five individual symptoms, patients are asked to report the extent to which they have experienced each symptom.

Due to the distribution of symptoms reported in our sample, we classified symptoms as “normal” (corresponding to “not at all” experiencing the symptom) and “any impairment” (all other responses). For subscales with multiple items, “any impairment” was assigned if impairment for any item in the scale was reported.

### Outcomes

Outcomes of interest were indicators of service utilization within three UCSF outpatient clinics specializing in colon cancer care: Gastrointestinal Oncology, Colorectal Surgery, and Cancer Survivorship.

Our primary outcome was the number of clinic visits (in-person or virtual) in the first 6 months after completing each QoL survey. Only visits with a licensed medical provider—including physician, nurse practitioner, registered nurse, or pharmacist—were included. Healthcare navigation visits (with a Healthcare Navigator or Medical Assistant) were excluded, as they are not considered clinical encounters. Secondary outcomes include the number of telephone encounters with clinical personnel and secure messages from patients concerning a medical issue. These data were extracted from our EMR in September 2022. Clinic visits and telephone encounters were identified by notes corresponding with these encounter types that were signed by a clinic provider. Messages with a medical question were identified by “patient medical advice request” categorization.

### Analyses

Our primary analyses assessed the association between self-reported symptoms and number of clinic visits (including surveillance visits) with a licensed medical provider within the Gastrointestinal Oncology, Colorectal Surgery, and Cancer Survivorship practices in the following first 6 months after each survey administration. Analyses were repeated for telephone encounters and patient messages as secondary outcomes.

We used generalized linear mixed regression model for repeated measurements with random trajectory for each participant to estimate adjusted risk ratios (RR) and 95% confidence intervals (CI) for the associations between symptoms and utilization count outcomes in each 6-month period following each QoL survey. The models adjusted for demographic characteristics (age, gender, race/ethnicity), clinical and surgical factors (e.g., stage, whether patient received surgery at UCSF, time since surgery, primary surgery grouping (Appendix Table 3), whether patient had received treatment or had a recurrence during follow-up), and whether the 6-month period after each exposure assessment was before or after March 2020 when the World Health Organization declared COVID-19 a pandemic and healthcare utilization initially declined before eventually rebounding [18, 19]. The repeated measures approach allowed participants with some missing data to contribute to the effect estimate; participants contributed information for each 6-month period for which they had non-missing exposure data.

We conducted six sensitivity analyses in which we (1) included clinical comorbidities as covariates; (2) included an indicator for missing QoL survey; (3) excluded patients with stomas, as stoma presence puts patients at risk of increased healthcare utilization [20–22]; (4) excluded time points when patients reported receiving active treatment; (5) censored patients with colon cancer recurrence at time of treatment re-initiation; and (6) excluded responses to QoL surveys after January 14, 2022 (and subsequent utilization), given there was less than 6 months of potential post-symptom utilization.

In addition, we examined the correlation between the patient-reported symptoms using Pearson’s correlation coefficients. For symptoms found to be significantly associated with clinic visits in models unadjusted for other symptoms, we conducted two post hoc analyses to determine if one or more of the symptoms was driving the observed associations. First, a composite variable was created defined as patients having at least one of these symptoms. The association between this composite variable and number of clinic visits was assessed. In the second, the significant symptoms were included in a single multivariable model to determine whether one (or more) was independently associated with number of clinic visits in the following 6 months.

Hypothesis tests were two-sided, and the significance threshold was set to 0.05. Statistical analyses were performed using SAS v9.4.

## Results

Among 126 patients (56% female, mean age 59) who met inclusion criteria, participants completed QoL surveys at a median of 4 time points (range 1–11) during follow-up. Appendix Fig. 1 includes QoL survey completion at each time point.

### Clinic visits

Patients averaged 1.2 clinic visits, 0.5 telephone encounters, and 3.2 patient-initiated messages during the first 6 months after reporting their symptoms at enrollment (Table 1). Adjusted for demographic characteristics, clinical and surgical factors, and timing relative to onset of the COVID-19 pandemic, patients with any abdominal pain (RR 1.45; 95% CI 1.15–1.83; *p* = 0.002), buttock pain (RR 1.30; 95% CI 1.00–1.68; *p* = 0.05), and increased stool frequency (RR 1.26; 95% CI 1.01–1.59; *p* = 0.05) had significantly more clinic visits in the 6 months after reporting their symptoms than those without these symptoms (Table 2). Patients with any other symptom examined (e.g., bloating, constipation, diarrhea, flatulence, fecal incontinence, or sore skin) did not have a significantly different number of clinic visits than those without the symptom.

**Table 1:** Characteristics and average 6-month post-symptom healthcare utilization of 126 colon cancer survivors at enrollment in the Lifestyle and Outcomes after Gastrointestinal Cancer (LOGIC) study (2017–2022)

Characteristic	Total	Mean visits, encounters, or messages within 6 months of baseline survey					
		Clinic visits		Telephone encounters*		Patient messages**	
		Mean (SD) ***	*p*-value	Mean (SD) ***	*p*-value	Mean (SD) ***	*p*-value
All patients, * n* (%)	126 (100)	1.16 (1.44)	–	0.51 (1.16)	–	3.21 (4.36)	–
Demographic characteristics							
Age, years, mean (SD)	59.0 (13.1)	–	–	–	–	–	–
Gender, * n* (%)							
Female	70 (55.6)	1.20 (1.58)	0.72	0.56 (1.28)	0.60	3.63 (4.53)	0.23
Male	56 (44.4)	1.11 (1.26)		0.45 (1.01)		2.70 (4.12)	
Race, * n* (%)							
American Indian or Alaska Native	3 (2.4)	1.00 (1.00)	0.20	1.00 (1.00)	**0.04**	1.33 (2.31)	0.10
Asian	18 (14.3)	1.28 (1.32)		0.56 (1.34)		4.00 (5.10)	
Black/African American	1 (0.8)	1.00 (–)		0.00 (–)		0.00 (–)	
White	92 (73.0)	1.05 (1.25)		0.40 (0.89)		2.93 (3.68)	
More than one race	6 (4.8)	1.00 (0.89)		0.33 (0.52)		2.00 (1.55)	
Unknown or not reported	6 (4.8)	2.67 (3.67)		2.00 (3.10)		7.83 (10.05)	
Ethnicity, * n* (%)							
Hispanic or Latino	12 (9.5)	1.08 (0.79)	0.98	0.75 (1.75)	0.69	3.33 (3.58)	0.96
Not Hispanic or Latino	113 (89.7)	1.17 (1.51)		0.49 (1.09)		3.21 (4.47)	
Unknown or not reported	1 (0.8)	1.00 (–)		0.00 (–)		2.00 (–)	
Education, * n* (%)							
Grade school	2 (1.6)	1.50 (0.71)	0.76	0.00 (–)	0.86	2.50 (2.12)	0.73
High school or the equivalent (e.g., GED)	6 (4.8)	0.83 (0.75)		0.17 (0.41)		1.50 (2.35)	
Associate degree (2-year college)	13 (10.3)	0.85 (0.90)		0.38 (0.96)		2.46 (2.93)	
Bachelor’s degree (4-year college)	44 (34.9)	1.30 (1.47)		0.45 (0.95)		3.73 (4.63)	
Graduate/professional	58 (46.0)	1.21 (1.61)		0.66 (1.42)		3.36 (4.68)	
Trade/vocational school	1 (0.8)	0.00 (–)		0.00 (–)		0.00 (–)	
Some college	2 (1.6)	0.00 (–)		0.00 (–)		0.00 (–)	
Clinical characteristics							
Body mass index, kg/m^2^, mean (SD)	25.7 (5.7)	–	–	–	–	–	–
Smoking status, * n* (%)							
No	79 (62.7)	1.09 (1.45)	0.55	0.57 (1.27)	0.84	3.43 (4.61)	0.56
Past	42 (33.3)	1.33 (1.49)		0.43 (1.02)		2.83 (4.03)	
Current	3 (2.4)	1.33 (0.58)		0.33 (0.58)		5.00 (2.65)	
Number of comorbidities, * n* (%)							
0	34 (27.0)	1.56 (1.86)	0.25	0.68 (1.43)	0.42	3.74 (4.93)	0.41
1	35 (27.8)	1.11 (1.75)		0.60 (1.38)		3.89 (5.33)	
2	23 (18.3)	0.83 (0.72)		0.17 (0.49)		2.52 (2.66)	
3 +	34 (27.0)	1.03 (0.83)		0.47 (0.93)		2.47 (3.48)	
Disease characteristics, *N* (%)							
Stage at diagnosis							
Stage I	17 (13.5)	0.65 (0.61)	**0.01**	0.35 (1.06)	0.23	2.35 (3.55)	**0.01**
Stage II	35 (27.8)	1.17 (0.89)		0.23 (0.49)		2.03 (2.53)	
Stage III	57 (45.2)	1.25 (1.55)		0.63 (1.41)		4.12 (4.87)	
Stage IV	10 (7.9)	2.30 (2.71)		1.10 (1.37)		5.80 (6.55)	
Unknown	7 (5.6)	0.00 (–)		0.43 (1.13)		0.14 (0.38)	
Metastasis							
Yes	22 (17.5)	1.82 (2.22)	**0.02**	0.95 (1.46)	**0.05**	5.18 (5.84)	**0.02**
No	104 (82.5)	1.02 (1.19)		0.41 (1.08)		2.80 (3.89)	
Treatment characteristics, * n* (%)							
Surgery performed at UCSF							
Yes	95 (75.4)	1.26 (1.46)	0.66	0.65 (1.20)	0.45	3.65 (4.29)	0.53
No	31 (24.6)	1.13 (1.45)		0.46 (1.16)		3.07 (4.40)	
Primary procedure grouping							
Right/transverse	50 (39.7)	1.04 (1.47)	0.83	0.34 (0.98)	0.57	2.60 (4.51)	0.53
Left/sigmoid	51 (40.5)	1.18 (1.28)		0.57 (1.28)		3.49 (3.81)	
Low pelvis	16 (12.7)	1.31 (2.02)		0.75 (1.18)		4.31 (6.05)	
Total/subtotal	9 (7.1)	1.44 (1.13)		0.67 (1.41)		3.11 (2.85)	
Time from surgery to enrollment							
Less than 6 months	23 (18.3)	1.43 (1.27)	**0.001**	0.70 (1.18)	0.20	4.26 (4.29)	**0.001**
6 months to 2 years	42 (33.3)	1.67 (1.88)		0.71 (1.45)		4.93 (5.16)	
2 to 5 years	43 (34.1)	0.93 (1.01)		0.37 (1.00)		1.98 (3.36)	
Greater than 5 years	18 (14.3)	0.17 (0.51)		0.11 (0.47)		0.83 (2.28)	
Active treatment at enrollment							
Yes	5 (4.0)	3.00 (1.87)	**0.003**	1.00 (1.22)	0.34	8.20 (4.02)	**0.01**
No	121 (96.0)	1.08 (1.38)		0.49 (1.16)		3.01 (4.27)	

*p*-values calculated using chi-squared use for categorical variables, ANOVA used for continuous variables

Values in bold represent statistical significance at *p* < 0.05

*Includes only telephone encounters with clinical personnel

**Includes only secure messages from patients concerning a medical question

***Reflects average Gastrointestinal Oncology, Colorectal Surgery, and Cancer Survivorship utilization within 6 months of completing the baseline QLQ-CR29 survey

**Table 2:** Association between self-reported individual gastrointestinal symptoms and 6-month post-symptom healthcare utilization of 126 colon cancer survivors enrolled in the Lifestyle and Outcomes after Gastrointestinal Cancer (LOGIC) study

Symptoms	Clinic visits			Telephone encounters*			Patient messages**		
	RR	95% CI	*p*-value	RR	95% CI	*p*-value	RR	95% CI	*p*-value
Single symptom models***									
Abdominal pain	**1.45**	**1.15–1.83**	**0.002**	1.11	0.62–1.98	0.729	**1.65**	**1.20–2.27**	**0.002**
Blood and mucus in stool	1.33	0.96–1.85	0.088	**2.46**	**1.26–4.84**	**0.009**	1.29	0.86–1.93	0.223
Buttock pain	**1.30**	**1.00–1.68**	**0.050**	1.39	0.74–2.58	0.303	1.39	0.97–1.98	0.076
Bloating	1.10	0.87–1.40	0.433	1.29	0.77–2.17	0.338	0.99	0.75–1.31	0.947
Constipation	1.19	0.93–1.51	0.166	0.66	0.35–1.25	0.208	0.96	0.71–1.30	0.799
Diarrhea	0.98	0.75–1.28	0.873	1.05	0.58–1.90	0.868	0.88	0.63–1.22	0.447
Flatulence	1.09	0.84–1.41	0.521	0.92	0.56–1.53	0.757	0.87	0.64–1.19	0.389
Fecal incontinence	0.82	0.55–1.20	0.304	1.19	0.55–2.57	0.650	0.87	0.53–1.42	0.570
Sore skin (stoma or anal area)	1.12	0.85–1.48	0.415	1.40	0.78–2.52	0.263	1.23	0.87–1.75	0.246
Stool frequency	**1.26**	**1.01–1.59**	**0.046**	1.35	0.82–2.20	0.237	1.07	0.80–1.44	0.642
Final model****									
Abdominal pain	**1.36**	**1.06–1.74**	**0.016**						
Stool frequency	1.21	0.96–1.52	0.104						
Buttock pain	1.21	0.91–1.61	0.189						

Values in bold represent statistical significance at *p* < 0.05

*Includes only telephone encounters with clinical personnel

**Includes only secure messages from patients concerning a medical question

***Estimates from a generalized linear mixed-effect model with random-effects per person. Single symptom models are adjusted for age, gender, race, ethnicity, stage, whether patient received surgery at UCSF, time since surgery, primary surgery grouping, whether patient had received treatment or had a recurrence during follow-up, and timing of post-exposure period relative to onset of COVID-19 pandemic

****Final model was further adjusted by symptoms found to be significantly associated with clinic visits in single-symptom models (i.e., abdominal pain, stool frequency, and buttock pain)

Correlations between self-reported symptoms were low to moderate (Appendix Table 4). Abdominal pain with buttock pain (*r* = 0.54) and bloating (*r* = 0.51) had the highest correlation coefficients. When considering abdominal pain, stool frequency, and buttock pain as a composite variable, patients with at least one of these symptoms had 38% more clinic visits (RR 1.38; 95% CI 1.09–1.73; *p* = 0.007) in the 6 months after reporting their symptoms than those who did not report any of these symptoms (Appendix Table 5). Patients with one of these symptoms had 25% more clinic visits (RR 1.25; 95% CI 0.98–1.60; *p* = 0.07) and those with at least two of these symptoms had 71% more clinic visits (RR 1.71; 95% CI 1.26–2.30; *p* = 0.001) than those who did not report any of these symptoms. Including all three symptoms as covariates revealed that abdominal pain was the only symptom that was independently associated with clinic visits; patients with abdominal pain had 36% more clinic visits (RR 1.36; 95% CI 1.06–1.74; *p* = 0.02) than those who did not report this symptom in multivariable models.

### Clinical telephone encounters

Adjusted for demographic characteristics, clinical and surgical factors, and timing relative to onset of COVID-19, patients with any blood or mucus in stool had significantly more clinical telephone encounters in the 6 months after reporting their symptoms than those without these symptoms (RR 2.46; 95% CI 1.26–4.84; *p* = 0.009). Patients with any other symptom did not have a significantly different number of clinical telephone encounters than those without the symptom.

### Patient-initiated messages

Adjusted for demographic characteristics, clinical and surgical factors, and timing relative to onset of COVID-19, patients with any abdominal pain had significantly more patient-initiated messages in the 6 months after reporting their symptoms than those without abdominal pain (RR 1.65; 95% CI 1.20–2.27; *p* = 0.002). Patients with any other symptom did not have a significantly different number of patient-initiated messages than those without the symptom.

### Sensitivity analyses

Our results were not materially changed when we (1) included clinical comorbidities as covariates; (2) included a missing indicator in the model representing when a QOL survey was missing; (3) excluded 7 patients with stomas; (4) excluded 11 QoL survey responses when patients were receiving treatment at time of completion; (5) censored 10 patients with colon cancer recurrence at time of treatment re-initiation; and (6) excluded responses to the QoL surveys after January 14, 2022 (and subsequent utilization).

## Discussion

In this prospective longitudinal cohort study of colon cancer survivors, we assessed the relationship between GI symptoms and utilization of healthcare services within settings that specialize in colon cancer care. In so doing, we identified that patients who experienced specific GI symptoms such as abdominal pain were at-risk of being high utilizers of certain services. This observation can inform strategies to reduce symptom burden and increase the effectiveness of healthcare usage. To our knowledge, this is the first study among colon cancer survivors to analyze GI symptoms in relation to future healthcare utilization [11, 12]. Our results revealed several important findings, which translate into actionable recommendations for practitioners who care for colon cancer survivors.

Firstly, abdominal pain, increased stool frequency, and buttock pain were predictive of more clinic visits. Patients who noted at least one of these three symptoms had 38% more clinic visits than those who denied all three symptoms, with presence of more of these symptoms associated with increasingly more clinic visits. These findings are likely attributed to numerous factors, which may include patient pursuit of symptomatic relief, patient fear of recurrence, and motivation of specialists in these settings to evaluate patients for recurrence and manage late physical effects [23–25]. Though a portion of colon cancer survivors are symptomatic at the time of recurrence, some providers deliver care that departs from evidence-based surveillance guidelines based on patient requests and fear of litigation, leading to overuse of healthcare services [25, 26]. These data offer medical practitioners preliminary survivorship recommendations that may affect future utilization and thus avoid unnecessary care. First, in addition to recommending evidence-based surveillance care that includes clinic visits, laboratory testing, colonoscopies, and other imaging studies, providers may offer strategies to minimize or prevent these symptoms altogether [27]. For example, we previously reported in this cohort of colon cancer survivors that patients who more closely followed the American Cancer Society nutrition and physical activity guidelines—in particular those who limited consumption of red or processed meat and maximized the variety of unique fruits and vegetables consumed—had lower odds of impaired stool frequency [15]. For patients at risk of this symptom, providers may emphasize the role of these guidelines via discussion during a visit or providing information in the After Visit Summary (AVS) or via the patient portal. Second, providers may offer patients anticipatory guidance and proactive recommendations for management of symptoms should they arise, which may be tailored to the timing of the symptoms after surgery. Prior studies have shown that cancer survivors desire supportive care for physical symptoms and have a positive attitude toward self-management [28]. Having contingency treatment plans, such as heat therapy or dietary modifications for abdominal pain, may empower patients to self-manage and avoid clinic visits, and having evidence-based areas of focus for this population would allow providers to maximally address concerns during time-limited visits [29]. Third, providers may focus on this constellation of symptoms (abdominal pain, increased stool frequency, and buttock pain) for early identification of, and proactive follow-up with, patients at-risk for more clinic visits. This could include proactive nurse telephone outreach or patient messaging, which may reduce the need for more intensive clinic visits. This strategy has been successful in other settings, such as in primary care or the management of chronic conditions, and may be beneficial for cancer survivors [30–33].

Given the recent trend toward virtual care catalyzed by the COVID-19 pandemic, it is critical to consider whether GI symptoms impact telephone visits and patient messaging [11, 12]. In contrast to the constellation of symptoms associated with more clinic visits, only blood or mucus in stool was predictive of increased telephone encounters, and only abdominal pain was predictive of increased patient messages. As blood in stool is a common presenting symptom among patients diagnosed with colon cancer, it is perhaps not surprising that colon cancer survivors who note the presence of blood (or mucus) in stool are quick to call a specialist with their concerns [34]. While further examination is needed to better understand this relationship and whether this results in ED visits or clinic visits, a particular focus on providing anticipatory guidance and education on discharge as part of survivorship care may be a meaningful interim strategy.

Importantly, of all symptoms evaluated, abdominal pain had the greatest association with clinic visits and was the only symptom associated with higher utilization of multiple forms of healthcare services (clinic visits and patient messages). This suggests that abdominal pain may be the most salient predictor of specialty care utilization among this population. Interestingly, among the general population, abdominal symptoms are among the most common reasons for primary care visits, with abdominal pain being the leading gastrointestinal symptom prompting an outpatient clinic visit [35, 36]. While colon cancer survivors are distinct from the general population, these individuals may be primed to seek care for this particular symptom, making anticipatory guidance and contingency recommendations particularly useful.

Finally, patients in our study who reported issues of peri-anal or peri-stomal sore skin had more clinic visits, telephone encounters, and patient messages, but these findings were not significant. Prior literature suggests that these symptoms are associated with increased clinic visits among patients with prior colorectal cancer diagnoses [9, 10]. The difference in our findings may be due to inherent differences in study design, including our focus on colon cancer patients vs. others including colon and rectal cancer patients, our longitudinal study design, and our use of EMR to determine utilization (vs. self-reported information) [9, 10]. They may alternatively be due to differences in availability and utilization of local resources outside the three modalities of care we examined, such as home health care services, which are readily available for patients with stomas who receive care at UCSF. It may also be that our study is underpowered to detect a significant difference, warranting confirmation of our findings through a larger study.

While our study expands on predictors of increased healthcare utilization among colon cancer survivors, it has limitations. First, this single-center study may limit generalizability of the results to other settings. Furthermore, this study excludes care received outside of the three specified clinics or at a non-UCSF healthcare facility. However, patients visit their primary care physician most often for non-cancer-related reasons while specialists are consulted for cancer care [9]. Nonetheless, our findings should be confirmed in a setting where all patient utilization is captured, such as an integrated health care delivery system. Second, our sample size precluded a sub-analysis of patients with very recent surgery (e.g., < 6 months before enrollment), who may have different utilization patterns from patients whose surgery was more remote. However, time since surgery was included as a covariate in our models, thus accounting for differences in utilization attributed to surgery timing. Third, our outcomes were limited to non-emergent outpatient services (i.e., excluding Emergency Department visits or inpatient admissions due to the small number of these encounter types within our study population). We are also not able to differentiate whether the patient visits or contacts were due to routine care versus a new symptom or symptom change and moreover whether the symptom was related to the patient’s cancer or another etiology. Our findings were still informative for predicting our outcome of interest. Fourth, our outcome variables were based on volume of visits, phone calls, and secure messages without any filter for the content of the encounter. While these encounters may not have been related directly or specifically to a GI symptom, the objective of our study was to assess the association of GI symptoms with overall utilization rather than utilization that was specifically related to those symptoms. Finally, while our study suggests that utilization did not differ by education level (which we consider a proxy for socioeconomic status), more than 80% of our study population had at least a bachelor’s degree. Further work to capture other patient factors such as insurance status, health literacy, or income is needed to understand utilization in a more educationally and socioeconomically diverse sample.

Future directions for this research include studying a broader range of patients with GI cancer, such as rectal or anal cancers, as well as using emerging technologies such as large language models to analyze electronic health record data to predict healthcare utilization among cancer survivors [37].

## Conclusions

Our findings identify GI symptoms associated with increased use of oncologic specialty care in colon cancer survivors, with abdominal pain as a particularly salient predictor of utilization in the following 6 months. While these are observational data, preemptive counseling of colon cancer survivors experiencing GI symptoms may improve patient education for postoperative and survivorship care and potentially avoid overuse of healthcare services and improve patient quality of life.

## Supplementary Information

Below is the link to the electronic supplementary material.

Supplementary file1 (DOCX 33.1 KB)

## Figures and Tables

**Fig. 1:**
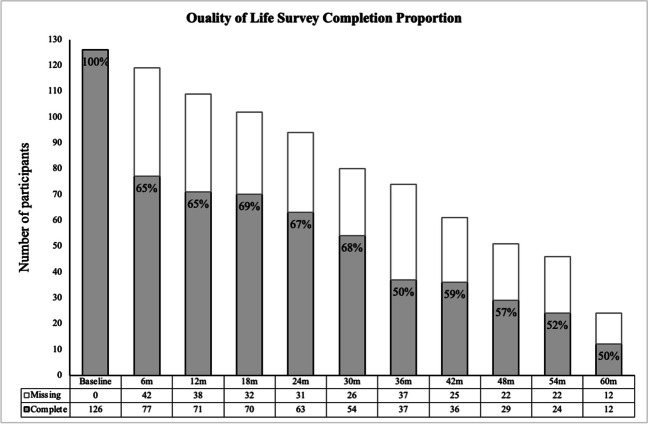
Completion proportion for the Quality of Life surveys among colon cancer survivors in the LOGIC study

## Data Availability

The data that support the findings of this study are not openly available due to reasons of sensitivity and are available from the corresponding author upon reasonable request. Data are located in controlled access data storage at University of California San Francisco.

## Appendix 1

**Table 3:** Primary surgery grouping definitions

Grouping	Primary surgery
Right/transverse	Right hemicolectomy
	Extended right colectomy
	Transverse hemicolectomy
Left/sigmoid	Left hemicolectomy
	Extended left colectomy
	Sigmoid colectomy
Low pelvis	Low anterior resection
	Low anterior resection with diverting loop ileostomy
Total/subtotal	Total proctocolectomy with end ileostomy
	Total proctocolectomy with ileal pouch-anal anastomosis
	Total colectomy with ileorectal anastomosis
	Total abdominal colectomy with end ileostomy
	Subtotal colectomy with ileosigmoid anastomosis

## Appendix 3

**Table 4:** Results of Pearson’s correlation between symptoms at baseline

Symptom	Pearson’s *r* (*p*-value)									
	AP	BMS	BP	Bl	Co	Di	Fl	FI	SS	SF
Abdominal pain [AP]	1.0	0.36 (< 0.01)	0.54 (< 0.01)	0.51 (< 0.01)	0.09 (0.31)	0.14 (0.13)	0.22 (0.02)	0.31 (< 0.01)	0.30 (< 0.01)	0.44 (< 0.01)
Blood and mucus in stool [BMS]		1.0	0.34 (< 0.01)	0.19 (0.03)	− 0.04 (0.63)	0.01 (0.92)	0.06 (0.50)	0.32 (< 0.01)	0.30 (< 0.01)	0.37 (< 0.01)
Buttock pain [BP]			1.0	0.44 (< 0.01)	0.33 (< 0.01)	0.02 (0.82)	0.08 (0.38)	0.35 (< 0.01)	0.38 (< 0.01)	0.45 (< 0.01)
Bloating [Bl]				1.0	0.30 (< 0.01)	0.06 (0.50)	0.20 (0.02)	0.37 (< 0.01)	0.10 (0.26)	0.38 (< 0.01)
Constipation [Co]					1.0	0.10 (0.26)	0.14 (0.12)	0.32 (< 0.01)	0.03 (0.74)	0.08 (0.36)
Diarrhea [Di]						1.0	0.21 (0.02)	0.07 (0.47)	0.27 (< 0.01)	0.29 (< 0.01)
Flatulence [Fl]							1.0	0.25 (< 0.01)	0.12 (0.19)	0.22 (0.01)
Fecal incontinence [FI]								1.0	0.36 (< 0.01)	0.45 (< 0.01)
Sore skin (stoma or anal area) [SS]									1.0	0.35 (< 0.01)
Stool frequency [SF]										1.0

## Appendix 4

**Table 5:** Results of post hoc analysis estimating the combined association between multiple gastrointestinal symptoms and number of clinic visits in the subsequent 6 months

Symptoms	Clinic visits		
	RR	95% CI	*p*-value
Models with composite symptom variable*			
Binary			
None of the three symptoms	1.0 (ref.)	1.0 (ref.)	1.0 (ref.)
At least one of the three symptoms	1.38	1.09–1.73	0.007
Ordinal			
None of the three symptoms	1.0 (ref.)	1.0 (ref.)	1.0 (ref.)
One of the three symptoms	1.25	0.98–1.60	0.073
Two or more of the three symptoms	1.71	1.26–2.30	0.001

*Composite variable of exposures found to be significantly associated with clinic visits in primary, single-symptom models (i.e., abdominal pain, stool frequency, and buttock pain)
